# Assessment of the effectiveness of podiatric nail cleaning in terms of patient health and quality of life: prospective observational study

**DOI:** 10.3389/fmed.2026.1750999

**Published:** 2026-03-30

**Authors:** Faustyna Kuros, Dominika Wcisło-Dziadecka

**Affiliations:** 1 Department of Medicine and Health Sciences, Institute of Health, Academy of Applied Sciences, Nowy Targ, Poland; 2 Department of Practical Cosmetology and Skin Diagnostics, Faculty of Pharmaceutical Sciences in Sosnowiec, Medical University of Silesia, Katowice, Poland

**Keywords:** deformity of nails, nail diseases, onychodystrophy, podiatry, primary health care

## Abstract

**Objective:**

Onychodystrophies constitute the most common nail diseases at any age. The condition is chronic and requires treatment due to the patient’s discomfort. Destructive changes to the nail apparatus are unaesthetic, often physically painful and socially embarrassing. The aim of the study was to focus on to evaluate the efficacy of nail cleaning with the use of milling cutters in improving the clinical picture of the nails, regardless of systemic comorbidities.

**Methods:**

This prospective observational study involved 50 patients diagnosed with the pathological condition of toenail dystrophy. The research was conducted between June 2021 and April 2024. The study used a method of cleaning the toenails with specialized sterile tools and milling cutters using a podiatric milling machine. Patients enrolled in the research project did not receive any other additional general or local treatment in the evaluated area. Procedures and follow-up visits were performed every 4 weeks until complete healing. Attention was also paid to the predispositions contributing to the occurrence of toenail plate deformities, as well as comparing the effectiveness of the therapeutic method used in terms of the type of defect, age and gender of the probands.

**Results:**

74.0% were women. The mean age was 41.80 ± 18.28 years. Nail plate degeneration (onychodystrophy) dominated among the types of nail deformity (70.0%). The mean length of therapy was 4.75 ± 2.96 months (ranging from 1 to 15 months). The predominant length of therapy was 4–5 months (30%). The evaluation of the effectiveness of therapy in the analyzed group amounted to 8.88 ± 1.26.

**Conclusion:**

Cleaning with sterile milling cutters was associated with a reduction in pain and an improvement in the clinical condition of the nail apparatus. The therapy allows for a safe and conservative approach to treating toenail dystrophy, improving patients’ overall wellbeing and quality of life.

## 1 Introduction

Nail deformities and dystrophies (onychodystrophies) are two broad terms that encompass all pathological deformities and changes in the nail apparatus (NA). These degenerations can be congenital or acquired up to traumatic conditions that impede the normal function of the NA matrix and bed, while affecting the quality of life of patients (1, 2). Many cases of onychodystrophies involve partial or complete damage to the various keratin layers of the toenail plate which arise spontaneously and are idiopathic in origin. Some dystrophies are common in children and adolescents, whereas others are more common in the older adults. In addition to a change in color (toenail dyschromia), degeneration can manifest as changes in the shape, structure, size, direction of growth, and thickness of toenails (2, 3).

The literature reports that dystrophic nails can be caused by a huge variety of diseases such as: fungal and non-fungal infections, viral infections, allergies, various non-infectious inflammatory dermatological diseases of the nail unit and tumors. In addition, many dermatological diseases, including lichen planus, spot baldness (alopecia areata), Darier’s disease, and psoriatic arthritis (PA), affect NA abnormalities. Various systemic, hepatic, and lung diseases are also associated with nail apparatus lesions (4–6). Certain drugs are also associated with PA dystrophy, because they interact with rapidly proliferating internal organs. Antibiotics, anticancer drugs, and antiretroviral drugs are also involved in the destruction of the toenail plates. Induced changes in the matrix, proximal nail fold, nail bed, hyponychium, or underlying bony phalanges secondarily cause abnormal nails. Onychodystrophy is chronic and requires treatment owing to patient discomfort. Destructive changes to NA are unaesthetic, often physically painful, and socially embarrassing (4, 6–8).

## 2 Materials and methods

### 2.1 Ethical considerations

This study was approved by the relevant Bioethics Committee of the Medical University of Silesia in Katowice (no. 18.05.2021, ID no. PCN/022/KB1/43/21). The study was conducted in accordance with the Declaration of Helsinki. All study participants (patients) provided written informed consent before participating in the medical research experiment. In minor participants, consent was given in writing by the legal guardian or parent of the participant.

### 2.2 Limitations

The study was conducted as a prospective observational, based on a comparison between baseline and post-therapy. Due to the fact that there is no recognized effective comparison therapy, as well as for ethical reasons, a control group without intervention was not established. This approach is widely used and accepted in studies involving aesthetic-functional therapies, where subjective patient evaluation constitutes an important component of efficacy. In this context, the fact that there is no control group not only preserves the scientific value of the study, but also aligns with the principles of clinical ethics. Direct comparisons with untreated patients or those receiving alternative interventions were not possible, which limits the ability to attribute the observed improvements solely to the studied procedure. The absence of a control group also restricts causal inference and requires that the reported outcomes be interpreted as associations rather than definitive evidence of treatment efficacy.

Additionally, as with all observational studies, the potential influence of unmeasured confounding factors cannot be fully excluded. Although the findings indicate a reduction in pain and an improvement in the clinical condition of the nail apparatus following cleaning with sterile milling cutters, these results should be interpreted with caution. Further studies, particularly controlled and randomized clinical trials, are warranted to confirm these observations and to more precisely determine the therapeutic effectiveness of the procedure.

A limitation of this study is that subjective outcomes, including nail appearance, comfort, and perceived pain, were assessed using an author-developed questionnaire specifically designed for clinical podiatry. This instrument was not formally psychometrically validated; therefore, results related to quality-of-life improvements should be interpreted cautiously and considered exploratory.

### 2.3 Participants and study design

This prospective observational study was conducted in a podiatry office on a group of patients with dystrophic teonails from June 2021 and April 2024. The study included 50 patients diagnosed with a pathological condition in the form of non-infectious toenail dystrophy, 74% were women. The mean age was 41.80 ± 18.28 years, ranging from 13 to 88 years. Detailed characteristics of the patients’ education and occupation are shown in Table 1. Eligibility was based on physical examination and detailed patient history. The original questionnaire was used to evaluate the therapy. It consists of several sections concerning the type, location, and etiopathogenesis of dystrophy, pain complaints, and assessment of the patient’s quality of life before and after therapy. Subjective parameters, including nail appearance, comfort, and perceived pain, were assessed using a study-specific, author-developed questionnaire. This instrument was not formally psychometrically validated, and therefore results related to quality-of-life improvements should be interpreted cautiously as exploratory. The evaluation was supplemented by medical records, including photographs taken following completion of several stages of the intervention, conducted at fixed time points, which allowed the clinical and subjective perspectives to be juxtaposed. In this way, it was possible to analyze not only the direct effect, but also its durability and progression. Patients with NA deformities in the form of subungual hematomas, non-infectious onychodystrophy and nails with impaired growth were included in the study. Systemic diseases did not constitute an exclusion factor, as long as they did not directly affect the condition of the nail apparatus or the feasibility of the procedure itself. Indeed, the aim of the study was to focus on the local aspect of treatment, i.e., to evaluate the efficacy of nail cleaning with the use of milling cutters in improving the clinical picture of the nails, regardless of systemic comorbidities. The methodology of the study was designed to allow evaluation of the effects of local therapeutic procedure while maintaining maximum homogeneity in the very nature of nail lesions.

**TABLE 1:** Demographic characteristics of the study group.

Variable	Number of observations/ mean ± SD	% Of group/median (IQR)
N	50	100.0
Sex		
Female	37	74.0
Male	13	26.0
Age, years	41.80 ± 18.28	39.00 (26.25; 57.75)
Education		
Primary	8	16.0
Vocational	14	28.0
Secondary	6	12.0
Post-secondary	1	2.0
Higher	21	42.0
Occupation status		
Student (primary/secondary school)	4	8.0
University student	2	4.0
Pensioner	11	22.0
Professionally active	33	66.0
Accountant	4	12.1
Teacher	3	9.1
Salesman	3	9.1
Dental assistant	2	6.1
Construction worker	2	6.1
Hairdresser	2	6.1
Waitress/waiter	2	6.1
Cook	2	6.1
Photographer	1	3.0
Gravedigger	1	3.0
Housewife	1	3.0
Consultant	1	3.0
Beautician	1	3.0
Dressmaker	1	3.0
Speech therapist	1	3.0
Warehouseman	1	3.0
Lawyer	1	3.0
Entrepreneur	1	3.0
Receptionist	1	3.0
Farmer	1	3.0
Secretary	1	3.0

SD, standard deviation; IQR, interquartile range.

### 2.4 Inclusion and exclusion criteria

This, in turn, allowed a meaningful analysis of the efficacy of the applied technique. The exclusion criteria for the study included patients who were minors, under 10 years of age, pregnant women, mental illness, or other ailments that could impair cooperation with a specialist, and no informed consent from the patient.

### 2.5 Treatment procedure

The method of nail cleaning in the podiatrist’s office involved controlled and gradual removal of the altered structure of the NA dystrophy using a milling machine and cutters. During the procedure, sterile cutters and tools meeting international quality management standards ISO 9001: 2008 were used. Procedures were performed with a Gerlach Luna AT Micro milling machine with a dust absorption system (80–130 L/min), with an adjustable speed that can be modified according to indications or conditions (from 6,000 to 30,000 RPM). Steel, diamond and tungsten cutters of various shapes and diameters were used to remove the damaged part of the nail plate and reach the nail bed. The nail plate was cleaned in the proximal to distal direction at 12,000 RPM. The procedure began with disinfecting and softening the nails (1–2 min) with a ready-made solution with INCI composition: Aqua purificata, 0.1% undecylenamidopropyl-betaine, 0.1% polyaminopropyl-biguanide (poliheksanid). A tungsten carbide cutter with a standard cross cut (6 mm diameter, 15 mm high) was used to remove hard nail fragments. Next, the detached, dystrophic nail plate structures—including hyperkeratosis of the nail bed—were cleaned and removed, using cuticle clippers and a slot cylindrical cutter with transverse notches (diameter 1.8 mm). This cutter was also used to remove cuticle and dirt within the nail walls. After deep cleaning of the nails, the sensitive structures of the NA (nail bed, hyponychium, nail walls) were polished and matted with the use of a fine-grained rounded conical diamond cutter with a diameter of 4 mm. Following cleaning procedure, the NA area was disinfected using a superoxidized solution with antimicrobial activity, and covered with a soft, sterile dressing. The procedure was performed at intervals of 4–6 weeks without no interruption to routine patient management practices. Comfortable shoes and daily foot hygiene were also recommended.

### 2.6 Statistical analysis

Statistical analysis was performed using R software, version 4.1.2. Variable description depended on variable type: numeric or ordinal variables were presented with mean and standard deviation as well as median and interquartile range (IQR). Nominal variables were described by the number of observations and the proportion of replies in the group. Comparisons before and after treatment were performed using the paired *t*-test or Wilcoxon test. The dependence of treatment effectiveness on age, sex, and type of nail deformation was verified with the Mann-Whitney U test and Kruskal-Wallis test due to non-normal distributions. Pairwise comparisons were performed using Dunn’s test with Bonferroni’s adjustment. Normality of distribution for assessment of test assumptions was performed with the Shapiro-Wilk test and further verified with skewness and kurtosis. All statistical calculations were performed at a significance level of alpha = 0.05.

## 3 Results

### 3.1 Clinical assessment of nails before therapy

The detailed frequencies of the symptoms and ailments are described in Table 2. One person used other treatment methods (laser therapy). None of the participants declared that their problem was completely eliminated by the time of participation in the study. No one was confirmed to have used other therapies related to nails (Table 2). Details of the reasons for deformation are presented in Table 3.

**TABLE 2:** Clinical assessment of nails before therapy.

Variable	Number of observations/ mean ± SD	% Of group/median (IQR)
Deformation of nails—duration		
1–2 Months	6	12.0
3–5 Months	8	16.0
6 Months	1	2.0
1 Year	7	14.0
> 1 Year	28	56.0
Deformation of nails: > 1 year	7.93 ± 8.16	5.00 (3.00; 8.50)
Deformation of nails—location		
Right foot		
Big toe	37	74.0
II Toe	1	2.0
III Toe	0	0.0
IV Toe	0	0.0
V Toe	2	4.0
Left foot		
Big toe	44	88.0
II Toe	1	2.0
III Toe	1	2.0
IV Toe	1	2.0
V Toe	2	4.0
Clinical symptoms		
Hypertrophy and thickening of nails	25	50.0
Curvature of the nail plate(s)	25	50.0
Upwards growth of the nail plate	12	24.0
Partial lack of nail plate(s)	12	24.0
Hyperkeratosis of the nail bed	22	44.0
Splinter hemorrhages	7	14.0
Dark red or green-black nail plates	17	34.0
Milky white nail plates	8	16.0
Yellow or brown nail plates	30	60.0
Detachment of the nail plate	36	72.0
Transverse or longitudinal nail furrows	14	28.0
Matting of the nail plate	35	70.0
Acute pain	7	14.0
Chronic pain	7	14.0
Ailments		
Pain when pressing in footwear	27	54.0
Pulsing pain when making movements	3	6.0
Redness and swelling of the nail shaft	12	24.0
Brittleness and cracking of the nail plate	10	20.0
Peeling of the nail plate	7	14.0
Exudate from under the nail plate	6	12.0
Other	0	0.0
Physical activity	12	24.0
Mountain trekking*	3	25.0
Football*	3	25.0
Gym*	2	16.7
Running*	2	16.7
Motorcross*	1	8.3
Skitours*	1	8.3
Cross-country skiing*	1	8.3
Other type of treatment used so far**	1	2.0
Complete elimination of nails deformation	0	0.0
Any other type of treatment related to nails at the moment	0	0.0

SD, standard deviation; IQR, interquartile range.

* Proportion calculated in relation to all patients with physical activity (*n* = 12).

** Laser therapy.

**TABLE 3:** Details of the diagnosis.

Variable	Number of observations	% Of group
Deformation of nails—type		
*Subungual hematoma*	12	24.0
Nail growth disorder (*onychoatrophia*)	6	12.0
Degeneration of the nail plate (*onychodystrophy*)	35	70.0
Nails comorbidities		
Thickening of the nail plate (*pachyonychia*)	20	40.0
Clawed nails (*onychogryphosis*)	11	22.0
Turret toenail (*unguis retroflexux*)	4	8.0
Nail psoriasis (*psoriasis unguium*)	0	0.0
Exfoliation of the nail plate (*onychomadesis*)	5	10.0
*Onycholysis*	40	80.0
Deformation of nails—etiopathogenesis		
Injuries	42	84.0
Mechanical (crushing, pressure, impact)	35	70.0
Chemical (resins, formaldehyde, detergents)	12	24.0
Thermal (UV lamp, LED)	1	2.0
Orthopedic deformation	46	92.0
Metatarsal (I and V rays) spread	31	62.0
Flat-valgus foot	2	4.0
Ankle drop	1	2.0
Hammer toes *(digiti mallei)*	20	40.0
Clawed toes (*digiti mallei*)	6	12.0
Equestrian finger (*digitus superductus*)	2	4.0
Tailor’s finger (*digitus subductus*)	4	8.0
*Hallux valgus*	31	62.0
*Hallux rigidus*	1	2.0
*Hallux varus*	2	4.0
Shoes	47	94.0
Too small/tight	26	52.0
Mountain	4	8.0
Work shoes with metal front	4	8.0
Sport	15	30.0
High-heels	5	10.0
Systemic illness	13	26.0
Diabetes	2	4.0
Circulatory failure	7	14.0
Atherosclerosis	0	0.0
Obesity	3	6.0
Other*	7	14.0
Other reasons	36	72.0
Too long nails	24	48.0
Hybrid nail polish in full shoes	24	48.0
Post-operational deformation of nails	6	12.0
Medications**	10	20.0
Other***	12	24.0

*Including hypothyroidism (*n* = 2), rheumatoid arthritis (*n* = 2), hypertension (*n* = 1), polyneuropathy (*n* = 1), and reflux (*n* = 1).

**Including: Euthyrox 75 (*n* = 2), Pentasa (*n* = 1), Plaquenil (*n* = 1), Adeksa (*n* = 1), Chemotherapy (*n* = 1), Atoris (*n* = 1), Pregabalin (*n* = 1), Diclacduo (*n* = 1), Polprazole (*n* = 1), Olfen 75 (*n* = 1), Acard (*n* = 1).

*** Including: Morton’s foot (*n* = 5), varicose veins (*n* = 2), syndactyly of the fingers (*n* = 1), pituitary adenoma (*n* = 1), ulcerative colitis (*n* = 1), nail plate reconstruction (*n* = 1), improper shortening of nails (*n* = 1), pseudomonasaeruginosa infection (*n* = 1).

### 3.2 Life quality assessment before and after the treatment

**FIGURE 1:**
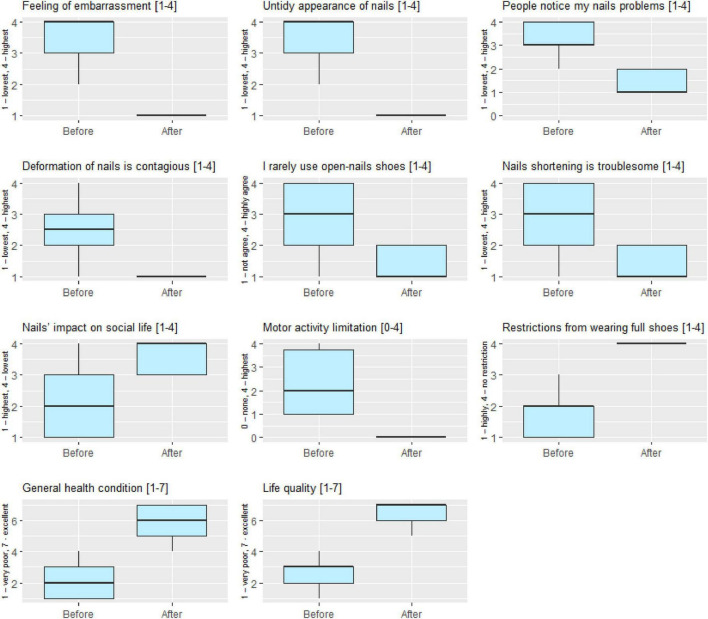
Boxplot charts presenting the distribution of life quality assessment scales before and after treatment.

Patients completed a series of life quality-related questions before and after treatment. All elements showed significant improvement after treatment (*p* < 0.001). We also evaluated whether the symptoms of nail deformation restricted the patients from wearing full shoes. Satisfaction from health condition grew from 2.02 ± 0.94 to 6.02 ± 0.94 on scale from 1 to 7. Life quality assessment grew from 2.56 ± 1.25 to 6.40 ± 0.70 on scale from 1 to 7 (Table 4). Outcomes reflecting changes in all measured life quality assessment scales were visualized as Figure 1.

**TABLE 4:** Comparison of life quality assessment before and after treatment.

Variable	Scale definition	Before	After	*p*
Feeling of embarrassment due to state of nails (1–4)	1—Lowest, 4—highest	3.44 ± 0.76	1.18 ± 0.39	**<0.001**
Worries about untidy appearance of nails (1–4)	1—Lowest, 4—highest	3.50 ± 0.61	1.20 ± 0.40	**<0.001**
Worries that other people notice my nails problems (1–4)	1—Lowest, 4—highest	3.18 ± 0.83	1.42 ± 0.50	**<0.001**
Worries that my deformation of nails is contagious and could spread (1–4)	1—Lowest, 4—highest	2.50 (2.00; 3.00)	1.00 (1.00; 1.00)	**<0.001^1^**
Irarely use open-nails type of shoes (1–4)	1—Not agree, 4—highly agree	3.00 (2.00; 4.00)	1.00 (1.00; 2.00)	**<0.001^1^**
Nails shortening is troublesome (1–4)	1—Lowest, 4—highest	2.98 ± 1.04	1.34 ± 0.48	**<0.001**
Nails’ impact on social life (1–4)	1—Highest, 4—lowest	2.00 (1.00; 3.00)	4.00 (3.00; 4.00)	**< 0.001^1^**
Motor activity limitation–Laitinen scale (0–4)	0—None, 4—highest	2.00 (1.00; 3.75)	0.00 (0.00; 0.00)	**<0.001^1^**
Symptoms restricting from wearing full shoes (1–4)	1—Highly, 4—no restriction	1.84 ± 0.89	3.96 ± 0.20	**<0.001**
General health condition (1–7)	1—Very poor, 7—excellent	2.02 ± 0.94	6.02 ± 0.94	**<0.001**
Life quality (1–7)	1—Very poor, 7—excellent	2.56 ± 1.25	6.40 ± 0.70	**<0.001**

Data are presented as mean and standard deviation or median and interquartile range. Comparisons of before and after were performed with paired *t*-test or Wilcoxon test^1^, as appropriate. Bold values indicate statistically significant *p*-values (*p* < 0.05).

### 3.3 Assessment of treatment effectiveness

The average therapy length was 4.75 ± 2.96 months (ranging, 1.00–15.00 months). The predominant therapy length was 4–5 months (30.0%). The average number of procedures was 4.08 ± 1.93, ranging from 1.00 to 10.00. The majority of patients underwent more than four procedures (60.0%). Pain level during procedures was assessed on a scale of 1–4, with an average outcome of 1.26 ± 0.44 on average. The patients assessed therapy effectiveness on a scale of 1–10. The average outcome in the analyzed group was 8.88 ± 1.26, ranging from 6.00 to 10.00 (Table 5).

**TABLE 5:** Assessment of treatment effectiveness.

Variable	Number of observations/mean ± SD	% Of group /median (IQR)
Therapy duration		
1–1.5 month	4	8.0
2–3 months	14	28.0
4–5 months	15	30.0
6 months	6	12.0
>6 Months	11	22.0
Therapy duration, months	4.75 ± 2.96	4.00 (3.00; 6.00)
Number of procedures		
1	1	2.0
2	10	20.0
3	8	16.0
4	1	2.0
>4	30	60.0
Number of procedures	4.08 ± 1.93	4.00 (3.00; 5.00)
Pain during procedure (1–4)*	1.26 ± 0.44	1.00 (1.00; 1.75)
Concerns during therapy		
I wasn’t afraid of anything	35	70.0
I was worried that I might damage my nail	9	18.0
I was afraid that I would have to go to podiatry for the rest of my life	1	2.0
Because of the therapy, I had to choose the right footwear	6	12.0
The whole therapy was burdensome for me	0	0.0
Other	0	0.0
Decision on therapy repeat if deformation of nails appeared again	50	100.0
Therapy impact on clinical picture of nails		
Is the structure of the nails smooth and clear?	50	100.0
Is the color of the nails pink, without discoloration?	48	96.0
Does the nail plate adhere to the nail placental?	44	88.0
Does the shape of the nail plate look natural?	43	86.0
Is the skin around the nails properly colored and unchanged?	49	98.0
Are there any pathological masses under the nail plate?	6	12.0
Does the nail plate grow back healthy?	49	98.0
Therapy effectiveness (0–10)**	8.88 ± 1.26	9.00 (8.00; 10.00)

SD, standard deviation; IQR, interquartile range.

* 1, no pain, 4 meant high pain.

**Therapy effectiveness was assessed on a scale from 0 to 10, where higher scores indicate greater effectiveness.

### 3.4 Effectiveness of the treatment—dependence on selected predictors

Age significantly determined the assessment of therapy effectiveness. Younger patients evaluated the therapy higher than older patients (median: 10.00 vs. 9.00 in scale from 0 to 10), MD = 1.00 CI_95_ [0.00; 1.00], *p* = 0.026. The therapy duration and number of procedures did not significantly depend on patient age (Table 6). Patients’ sex did not significantly determine effectiveness assessment, therapy duration, or number of procedures (*p* > 0.05) (Table 7).

**TABLE 6:** Effectiveness of treatment against age of patients.

Variable	Age < median (39 years)	Age ≥ median (39 years)	MD (95% CI)	*p*
Therapy effectiveness [0–10]	10.00 (8.50; 10.00)	9.00 (8.00; 9.50)	1.00 (0.00; 1.00)	**0.026**
Therapy duration, months	5.00 (3.50; 6.00)	3.00 (2.50; 4.00)	2.00 (0.00; 2.00)	0.073
Number of procedures	4.00 (3.50; 5.00)	4.00 (2.00; 4.00)	0.00 (0.00; 2.00)	0.097

Data are presented as median and interquartile range. MD, median difference (age < median vs. age ≥ median); CI, confidence interval. Comparisons between groups were performed using the Mann-Whitney U test. Bold values indicate statistically significant *p*-values (*p* < 0.05).

**TABLE 7:** Effectiveness of treatment against sex of patients.

Variable	Females	Males	MD (95% CI)	*p*
Therapy effectiveness (0–10)	9.00 (8.00; 10.00)	9.00 (7.00; 10.00)	0.00 (0.00; 2.00)	0.441
Therapy duration, months	4.00 (3.00; 6.00)	4.00 (3.00; 5.00)	0.00 (-1.00; 2.00)	0.712
Number of procedures	4.00 (3.00; 5.00)	4.00 (3.00; 5.00)	0.00 (-1.00; 1.00)	0.874

Data are presented as median and interquartile range. MD, median difference (females vs. males); CI, confidence interval. Comparisons between groups were performed using the Mann-Whitney U test.

**FIGURE 2:**
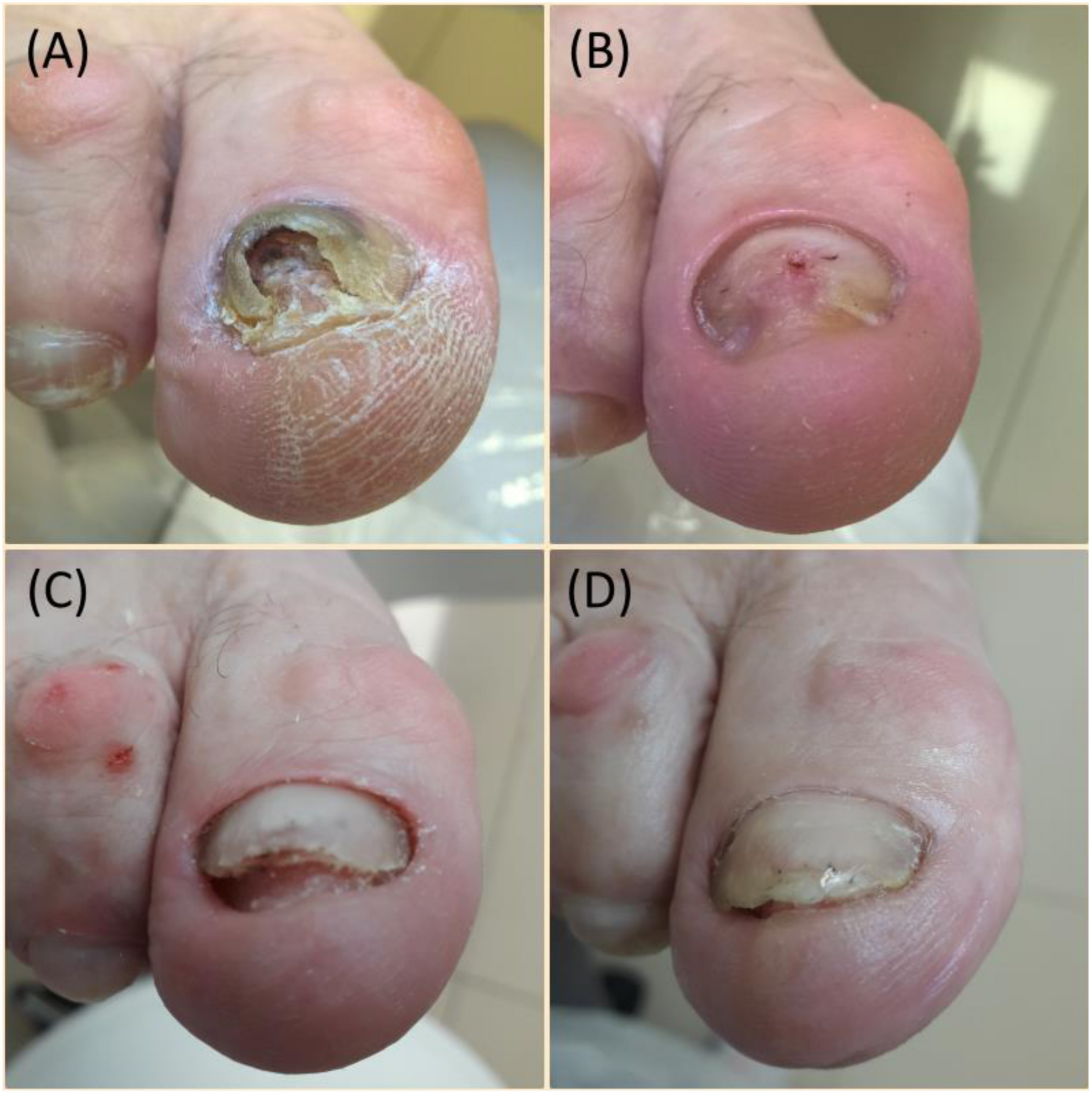
Nail plate degeneration (onychodystophia). Duration of disease 10 years: **(A)** cause of deformity: trauma, overweight, toenail deformities; **(B)** cleaning with milling cutters immediately after treatment; **(C)** toenail after 4th clearing; **(D)** effect after 12 months—9 treatments performed. Source: Own archives.

**FIGURE 3:**
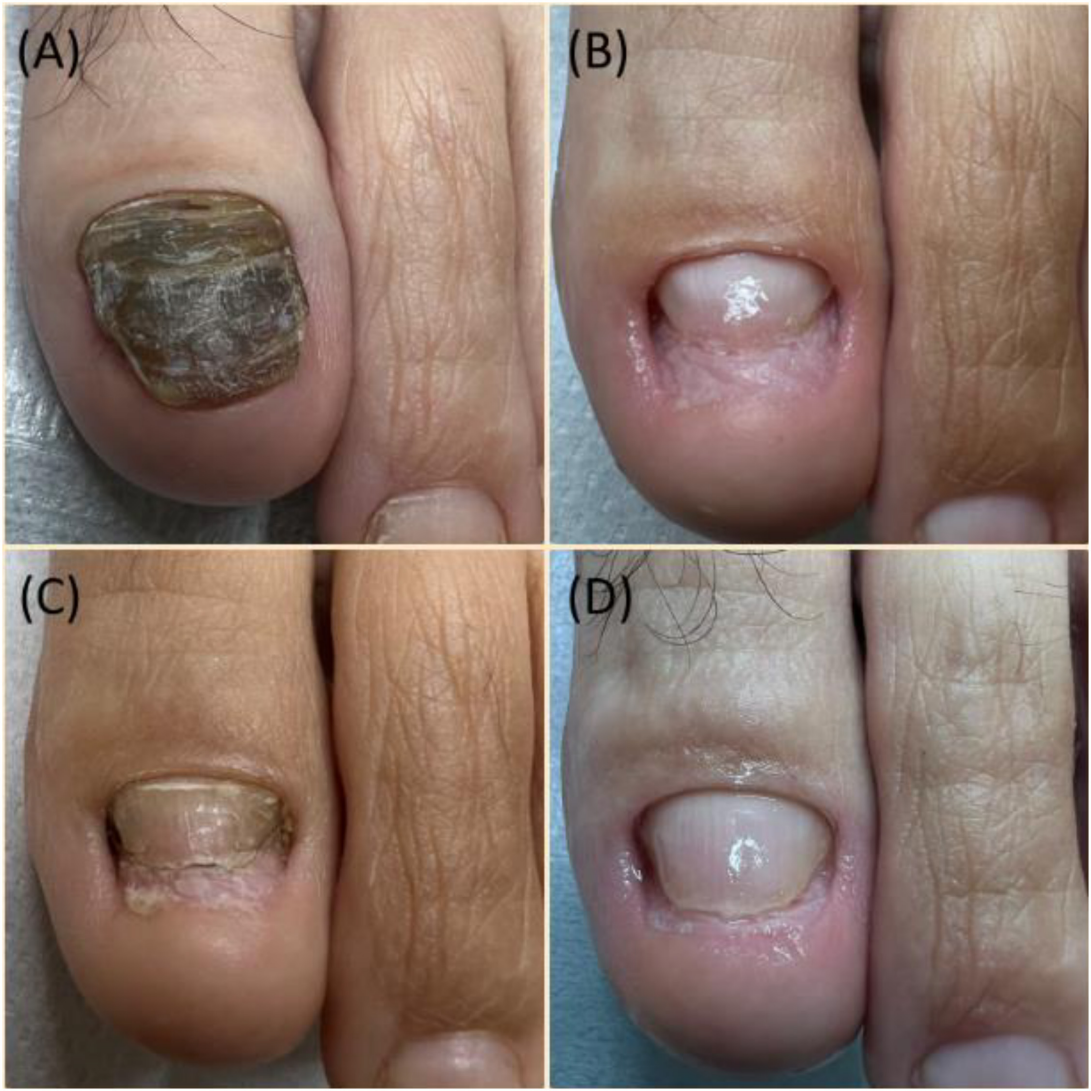
Clinical images showing nail apparatus degeneration in the form of onychogryphosis. **(A)** Initial condition with a 3-year history due to trauma, pain under shoe pressure, metatarsal separation, and hallux valgus deformity. **(B)** Condition immediately after treatment. **(C)** Condition during a 3-month therapy period. **(D)** Final effect after 5 months of therapy.

**FIGURE 4:**
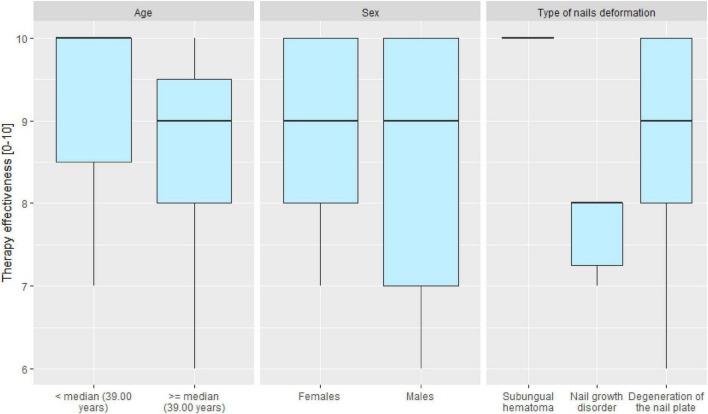
Boxplot chart presenting the distribution of therapy effectiveness assessment in scale from 0 to 10, for total group and in analyzed age, sex and type of deformation subgroups.

The type of deformation determines the assessment of therapy effectiveness. Patients with subungual hematoma type deformation assessed the therapy with the highest effectiveness, patients with degeneration of the onychodystrophy assessed the therapy lower, and patients with nail growth disorder (onychoatrophia) had the lowest score. The differences in therapy assessments were statistically significant (*p* = 0.005). Further pairwise comparisons indicated that the assessment of patients with subungual hematoma was significantly higher than that of patients with nail growth disorder (onychoatrophia) and the assessment of patients with subungual hematoma was significantly higher than that of patients with onychodystrophy. Therapy duration and number of procedures did not significantly depend on the type of deformation (Table 8). The effects of cleaning dystrophic nails of different etiologies are shown in Figures 2A–D, 3A–D. Therapy effectiveness in split to age groups, sex, and type of deformation was visualized(Figure 4).

**TABLE 8:** Effectiveness of treatment against type of deformation.

Variable	*Subungual hematoma*	Nail growth disorder *(onychoatrophia)*	Degeneration of the nail plate *(onychodystrophy)*	*p*
Therapy effectiveness (0–10)	10.00 (10.00; 10.00)^ab^	8.00 (7.25; 8.00)^a^	9.00 (8.00; 10.00)^b^	**0.005**
Therapy duration, months	4.00 (3.00; 8.00)	2.50 (2.00; 3.00)	4.00 (3.00; 6.00)	0.096
Number of procedures	4.00 (3.00; 6.00)	2.00 (2.00; 3.50)	4.00 (3.00; 5.00)	0.102

Data are presented as median and interquartile range. Comparisons between groups were performed using the Kruskal-Wallis test. *Post-hoc* comparisons were performed using Dunn’s test with Bonferroni adjustment (*^a^*p adj = 0.005, ^*b*^p adj = 0.029). Patients with more than one type of deformation (*n* = 3) were excluded from analysis. Bold values indicate statistically significant *p*-values (*p* < 0.05).

The present findings should be considered hypothesis-generating and warrant further investigation in prospective controlled studies.

## 4 Discussion

Dystrophic NA lesions are a common condition encountered in daily dermatological and podiatric practice. The differential diagnosis of the etiology of onychodystrophy is broad and poses a therapeutic challenge for specialists. The general appearance and clinical signs of the nail plates help diagnose the patient’s condition and should constitute an integral part of a detailed examination. Some nail diseases make the nail plate thicker or alter other NA structures. Deformities of the nails and the soft tissues around them often result from trauma to the nail area. They affect people who use their feet extensively and are a common complication of surgical procedures. During a clinical evaluation, it is important to ask the patient about any past nail injuries, types of footwear, occupational exposures or significant medical conditions. During the physical examination, the foot should be evaluated for structural abnormalities, especially for toe deformities, which cause morphological and functional disorders of the lower extremities in patients (9–11). Patients often complain they encounter difficulty in performing daily activities or pain, which affects the quality of their life. Treatment is difficult and long-term, due to the slow growth of toenails and resistance to therapy. Available therapies include topical and intracutaneous corticosteroids, tacrolimus ointment, systemic isotretinoin, laser therapy and surgical removal. Despite many efforts, clinical studies to date on the aforementioned methods have shown variable efficacy or inconsistent results. They can cause significant discomfort due to severe pain, and some patients choose not to undergo such invasive treatment (6, 10–12). An alternative method to laser therapy and other invasive treatments is mechanical nail plate debridement, which is designed to partially or completely remove the nail in cases of trauma, onycholysis, hematoma, infection or nail thickening. Several techniques for mechanical cleaning of nails are available, including filing (polishing block, file) or cutting (milling machine, cutters) also known as mechanical abrasion, which involves filing the nail plate to reduce its thickness or damage it completely. This procedure does not require anesthesia but also should not cause any pain or periungual damage (13).

In their review, Álvarez et al. (14) presented the use of a mechanical abrasion technique to treat nail deformities in which the nail bed has atrophied due to chronic onycholysis and other disorders associated with NA bed damage. Using a variable speed drill (with adjustable speed from 5,000 to a maximum of 22,000 RPM) and cylindrical granite, diamond and tungsten tips, the damaged nail plate, hyponychium and nail walls were removed. The final stage of treatment was to achieve pinpoint bleeding while treating directly the nail bed. The procedure was performed under local anesthesia with toe block. Alcohol was used during the procedure to keep the treated area clean and moist, and to control the temperature generated during the procedure. During the procedure, the authors took thermographic readings to assess the tissue response to the mechanical forces and generated heat. Depending on the speed of the drill bits and the tip material (granite, diamond, and tungsten), the tissue may reach high temperatures of 40–55 degrees Celsius (104–131 degrees Fahrenheit). According to the authors, the heating facilitates hyperkeratosis abrasion, biofilm cleaning (in the case of onychomycosis) and causes the disappearing nail bed (DNB) to regress, causing slight bleeding (14).

Adamczyk et al. (15) described treatment options for chronic onycholysis with DNB using combined conservative methods. The report describes the procedure performed in two patients (a 23-year-old woman and a 65-year-old man) with advanced damage to the nail apparatus. Each case required an individual approach, using one, two, or three described elements of therapy: nail bed cleaning, applying Onygen^®^ cream, massaging the nail bed, orthoses and kinesiotaping of nail walls. The key point of the therapy involved the use of several treatment methods with different effects on the nail apparatus. The results of the described therapy were highly satisfactory, the only drawback being its prolonged duration, which is due to the slow growth of toenails (15).

Another case report describes the case of a 62-year-old man who reported to a specialist clinic with post-traumatic onychodystrophy involving all 10 toenails for 20 years. The patient complained of discomfort when wearing shoes, difficulty trimming his nails and a sense of shame about the appearance of his nails. Physical examination revealed thickening of the nail plate and xanthomatous paronychia. Treatment with a topical 40% urea cream for 2 months proved ineffective. The patient began using a home nail drill twice a month, which effectively thinned his nails, allowing them to be cut efficiently, improving his quality of life and making him more comfortable when wearing shoes (9). According to Chiacchio et al., abrasion of the nails is the best treatment, especially in patients whose clinical condition does not allow for definitive surgery. Recommending the said method to the patient does not mean the patient will follow the instructions correctly. A specialist cleaning procedure in a doctor’s or podiatrist’s office would be more appropriate and safer (16).

According to evidence-based medicine (EBM) studies, NA dystrophies affect quality of patient’s life, including physical and social functioning and their emotional health. Patients often see a podiatrist for treatment of nail pain and changes in nail structure (nail trimming problems), difficulty walking, discomfort wearing shoes, pressure on the nails and embarrassment about their appearance. Treatment options such as nail avulsion, topical/transdermal corticosteroids, clobetasol and urea treatment often prove unsatisfactory. According to scientific reports, surgical removal of nails is considered a traumatic procedure commonly used, consequently leading to permanent dystrophy with onycholysis (9, 13–16).

In our study, non-infectious onychodystrophy (70.0%) and subungual hematoma (24.0%) constituted the predominant types of NA nail deformity. Onycholysis was present in 80.0% of the subjects, thickening of the nail plate was observed in 40.0%, and onychogryphosis in 22.0% of the study participants. Patients often indicated that the appearance of their nails could be the result of a mycotic infection; however, it is necessary to consider other factors, such as biomechanics and the type of footwear. Biomechanics describes both non-pathological and pathological forces involved in the gait cycle. Pathological forces can lead to toe deformities, soft tissue lesions (such as corns and calluses), hallux valgus, arch and heel pain, and even skin damage in neuropathic patients. Our study examined the influence of various factors on the occurrence of nail deformities. Analysis showed that 94.0% of participants wore footwear that could contribute to nail problems. Among the subjects, shoes that were too small or tight were the most common, affecting 52.0% of the group, suggesting that this is the main cause of nail dystrophy. Additionally, 92.0% of participants reported orthopedic deformities as the second most common cause, and injuries were the third most common cause, occurring in 84.0% of subjects. These results emphasize the significance of the analysis of biomechanics and proper footwear selection in the context of nail condition. We also assessed whether nail deformity symptoms prevented the patients from wearing full footwear. The mean score ranged from 1.84 ± 0.89 before treatment to 3.96 ± 0.20 after treatment, indicating that restrictions on wearing full shoes diminished after our podiatric therapy. After therapy, open-toed shoes were used more frequently (median 3.00 before therapy and 1.00 after therapy on a scale of 1–4). Nail shortening became less bothersome after therapy (from 2.98 ± 1.04 to 1.34 ± 0.48 on a scale of 1–4). Most subjects reported an improvement in the overall aesthetic appearance of the NA. The prevailing length of therapy was 4–5 months (30%). Most patients had more than four procedures performed (60%). The level of pain during the procedures was rated on a scale of 1– 4, with a mean score of 1.26 ± 0.44, which confirms that the discomfort experienced was minimal and the cleaning procedures performed with the cutters were well tolerated by the patients. The low level of pain may also indicate the efficacy of the used procedures, as well as their safety.

Evaluation of the quality of life before and after treatment showed significant improvement. Satisfaction with health condition increased from 2.02 ± 0.94 to 6.02 ± 0.94, and quality of life assessment increased from 2.56 ± 1.25 to 6.40 ± 0.70 (*p* < 0.001). Feelings of shame and concerns about the nail appearance diminished significantly, and concerns about the contagious nature of nail problems decreased from a median of 2.50–1.00. The impact of nail conditions on patients’ social lives was also lower (median increased from 2.00 to 4.00). Restrictions on physical activity diminish from a median of 2.00 before the therapy to 0.00 after the therapy. Therefore, it can be assumed that the patients were satisfied with the effects of the treatment, which may have contributed to their positive assessment of the overall aesthetic appearance of their nails. The observation period in this study was limited to a maximum of 15 months, with most therapies lasting 4–5 months. No recurrence of nail changes or reappearance of pain was observed during this time; patients were informed about the possibility of such events and monitored accordingly. Nevertheless, these findings should be interpreted cautiously, as the observation was largely limited to the intervention period. Further prospective studies with extended follow-up are needed to assess the durability of clinical improvement and to determine whether the observed effects represent lasting nail apparatus regeneration or require maintenance therapy.

The results of our own study are consistent with those of other authors, confirming the efficacy of mechanical cleaning (grinding) of nails with cutters, on the basis of previous reviews and clinical reports, which testify to a positive therapeutic effect. Limitations of the studies to date are very small sample sizes (case studies) or a homogeneous cohort of patients (9, 15–18). In our prospective single-arm study with the intervention, the fact that there is no control group may be considered a potential limitation, but it should be emphasized that this decision was conscious and based on both ethical and practical considerations. In the case of dystrophic and traumatic lesions of the nail plate, it was considered ethically unjustified to include a group devoid of treatment, despite the availability of a safe and potentially effective procedure. In addition, there is no clearly recognized therapy with a comparable profile of action in the studied area. The combination of subjective and clinical data, collected from medical records, specialist observations and pre- and post-intervention questionnaires, provides a reliable basis for evaluating the procedure efficacy. Although the study does not meet the criteria of a classic randomized design, its prospective observational approach reflects real-world clinical conditions, in which the therapeutic effect encompasses both medical and psychosocial aspects. In our study, therapy parameters evaluated in 50 patients improved significantly, with no adverse effects observed, and the removal of dystrophic and traumatic nail tissue by a podiatrist contributed to an improved clinical picture of the nail apparatus. To date, no randomized controlled trials have specifically addressed regular mechanical nail cleaning as an independent therapeutic intervention. However, randomized studies on related nail interventions (e.g., topical therapies for onychomycosis) provide useful insights but do not specifically address mechanical nail cleaning. Our prospective observational findings provide preliminary, real-world evidence on this procedure and its association with nail condition and patient comfort, highlighting the need for future controlled studies to confirm these effects.

## 5 Conclusion

Regular nail cleaning performed in a podiatrist’s office was associated with improvements in nail condition and aesthetics during the study period. The procedure appeared to be a safe and well-tolerated management option that may contribute to improved patient comfort.

However, due to the absence of a control group, these findings should be interpreted with caution. Further controlled studies, preferably randomized, are needed to confirm these observations.

## Data Availability

The original contributions presented in this study are included in the article/supplementary material, further inquiries can be directed to the corresponding author.
